# Genomic diversity and organization of complex polysaccharide biosynthesis clusters in the genus *Dickeya*

**DOI:** 10.1371/journal.pone.0245727

**Published:** 2021-02-11

**Authors:** Manish Ranjan, Devanshi Khokhani, Sanjeeva Nayaka, Suchi Srivastava, Zachary P. Keyser, Ashish Ranjan

**Affiliations:** 1 CSIR-National Botanical Research Institute (CSIR-NBRI), Lucknow, Uttar Pradesh, India; 2 Department of Bacteriology, University of Wisconsin-Madison, Madison, Wisconsin, United States of America; 3 Department of Plant Pathology, University of Minnesota—Twin Cities, St. Paul, Minnesota, United States of America; 4 Department of Agronomy, University of Wisconsin-Madison, Madison, Wisconsin, United States of America; 5 Department of Plant Sciences (SLS), University of Hyderabad, Hyderabad, India; Osmania University, INDIA

## Abstract

The pectinolytic genus *Dickeya* (formerly *Erwinia chrysanthemi*) comprises numerous pathogenic species which cause diseases in various crops and ornamental plants across the globe. Their pathogenicity is governed by complex multi-factorial processes of adaptive virulence gene regulation. Extracellular polysaccharides and lipopolysaccharides present on bacterial envelope surface play a significant role in the virulence of phytopathogenic bacteria. However, very little is known about the genomic location, diversity, and organization of the polysaccharide and lipopolysaccharide biosynthetic gene clusters in *Dickeya*. In the present study, we report the diversity and structural organization of the group 4 capsule (G4C)/O-antigen capsule, putative O-antigen lipopolysaccharide, enterobacterial common antigen, and core lipopolysaccharide biosynthesis clusters from 54 *Dickeya* strains. The presence of these clusters suggests that *Dickeya* has both capsule and lipopolysaccharide carrying O-antigen to their external surface. These gene clusters are key regulatory components in the composition and structure of the outer surface of *Dickeya*. The O-antigen capsule/group 4 capsule (G4C) coding region shows a variation in gene content and organization. Based on nucleotide sequence homology in these *Dickey*a strains, two distinct groups, G4C group I and G4C group II, exist. However, comparatively less variation is observed in the putative O-antigen lipopolysaccharide cluster in *Dickeya spp*. except for in *Dickeya zeae*. Also, enterobacterial common antigen and core lipopolysaccharide biosynthesis clusters are present mostly as conserved genomic regions. The variation in the O-antigen capsule and putative O-antigen lipopolysaccharide coding region in relation to their phylogeny suggests a role of multiple horizontal gene transfer (HGT) events. These multiple HGT processes might have been manifested into the current heterogeneity of O-antigen capsules and O-antigen lipopolysaccharides in *Dickeya* strains during its evolution.

## Introduction

The genus “*Dickeya*” (*D*.) is a newly formed group of six pectinolytic phytopathogenic species formerly classified as *Erwinia chrysanthemi* [1]⁠. Tuber soft rot and stem rot (Blackleg) caused by these phytopathogenic enterobacterales, *Dickeya* spp. are prevalent in the European countries [2]⁠. *Dickeya* species such as *D*. *dadantii* and *D*. *solani* are regarded as two of the top ten bacterial plant pathogens of economic and scientific significance [3]⁠. Several species of *Dickeya* cause soft rot in many economically important crops, with the most prominent ones affecting potato, maize, tomato, *Chrysanthemum* spp., banana, and *Dianthus* spp. [1, 4]⁠. The pathogenicity and the host range of these pectinolytic *Dickeya* depend on multi-factorial processes including adhesion, penetration, interaction with virulence factors like flagella, lipopolysaccharide, exopolysaccharide (EPS) and other effector proteins, and their multi-layer regulation [5]⁠.

Capsular polysaccharides (CPS) and lipopolysaccharides (LPS) are the major bacterial surface polysaccharides that continuously evolve to protect bacterial pathogens against bacteriophages [6, 7]. CPS are high molecular weight acidic polysaccharides that differ depending on the mechanism of their synthesis and assembly [8, 9]. Bacterial LPS are the major outer membrane surface components present in most Gram-negative bacteria. LPS typically consists of three parts: (a) a hydrophobic domain known as lipid A (or endotoxin) (b) a nonrepeating "core" oligosaccharide and (c) a distal polysaccharide chain (or O-antigen lipopolysaccharide). Lipid A, the most conserved hydrophobic part of LPS, consists of glucosamine disaccharide units with β (1→ 6) linkages. These sugars are linked to acylated fatty acid chains in the bacterial membrane. The core-oligosaccharide of LPS is divided into two main parts—the inner core and the outer core. The inner core consists of 3-deoxy-D-mannooctulosonic acid (Kdo) and heptose sugars, whereas the outer core consists of hexose sugars like D- glucose, D-mannose, D-galactose, etc. The O-antigen component is the most diverse part of LPS and provides a high degree of variability with specificity. Enteropathogenic and enterohaemorrhagic *E*. coli, a Gram-negative enterobacterium, forms a group 4 capsule (G4C)/ O-antigen capsule [10]. Compared to LPS, G4Cs are not attached to the bacterial surface via lipid but rather with specific surface proteins [11]. O-antigen modifications influence different pathogen infection stages, including colonization (adherence), and play a role in bypassing the host defense mechanism by masquerading host molecules in both plants and animals [12, 13]⁠. Both LPS and CPS also contribute to antibacterial defense by mitigating the effects of antimicrobial peptides [14]. These complex polysaccharides are key virulence factors in plant-pathogen interactions [15, 16]. Therefore, it is of utmost importance to determine their role in *Dickeya* host-pathogen interactions.

Bacterial soft-rot primary symptoms include pectin degradation of the middle lamella and primary plant cell wall caused by secreted pectinases [17]. These infected plant tissues appear as wet macerated foul-smelling rot. These symptoms are common to many different types of phytopathogenic bacteria. Therefore, focus on key virulence determinants and their genomic counterparts specific to the genus *Dickeya* is essential. LPS and CPS are key virulence determinants of many phytopathogenic bacteria, and their different components play a crucial role during pathogenesis [18–21]. Proper identification and classification of these virulence factors will guide the diagnosis and disease prevention in a systematic way.

In addition to the previous six classified *Dickeya* species, three more species are reported. *Dickeya solani* is a recent addition to the genus *Dickeya* as a taxonomically established species [22]. Additionally, two new species, "*Dickeya aquatica*” and “*Dickeya fangzhongdai*," were also reported [23, 24]. Phylogenetic classification based on multi-locus sequence analysis (MLSA) corroborates with whole genome-based average nucleotide identity (ANI) for species resolution [25]. Accounting for 54 strains of the genus *Dickeya*, we performed species delineation using criteria of RNA-polymerase beta subunit (*rpoB*) gene identity, whole genome-based species criteria of ANI, and whole genome based digital DNA-DNA hybridization (dDDH) method [26]. The *rpoB* gene is a promising phylogenetic marker as it is a single-copy gene in the bacterial genome and relatively immune to HGT [27]. Our study reflects on the classification of different species of *Dickeya* based on these species criteria and their co-occurrence with specific complex polysaccharide biosynthesis clusters. In this study, we provide comprehensive analyses of G4C or O-antigen capsule cluster diversity within the genus *Dickeya*. We also summarize the variation and organization of the conserved genomic islands that regulate the biosynthesis of G4C (OAg-capsule), O-antigen lipopolysaccharides (OAg-LPS), Enterobacterial Common Antigen (ECA), and other core lipopolysaccharide biosynthesis cluster in these strains along with their evolution among different species of *Dickeya*.

## Material and methods

### Bacterial strains data

A total of 54 genomes of *Dickeya* species are included in these analyses (Table 1). All the genomes were downloaded from the National Center for Biotechnology Information (NCBI) ftp server (ftp://ftp.ncbi.nlm.nih.gov/genomes, downloaded in March 2019) using their accession number as mentioned in Table 1.

**Table 1:** Group 4 capsule (G4C) coding gene cluster of different species in *Dickeya*.

SL. No	*Dickeya* Strains	G4C/O-antigen capsule cluster			NCBI Accession
		Size (Kb)	No. Of ORF	GC content (%)	
**G4C Group I**					
**1**	*D*. *dianthicola* GBBC 2039	22.3	20	52.5	AOOM01000000
**2**	*D*. *dianthicola* NCPPB 453	28.9	25	48.4	AOOB01000000
**3**	*D*. *dianthicola* DE440	28.9	25	48.4	PJJB00000000
**4**	*D*. *dianthicola* IPO 980	28.9	25	48.4	AOOS01000000
**5**	*D*. *dianthicola* ME23	28.9	25	48.4	CP031560
**6**	*D*. *dianthicola* RNS04.9	28.9	25	48.4	CP017638
**7**	*D*. *dianthicola* SS70	28.9	25	48.4	QESZ00000000
**8**	*D*. *dianthicola* WV516	28.9	25	48.4	PJJC00000000
**9**	*D*. *dianthicola* NCPPB 3534	28.9	25	48.4	AOOK01000000
**10**	*D*. *dadantii* NCPPB 898	31.3	27	48.9	AOOE01000000
**11**	*D*. *dadantii* DSM 18020	31.3	27	48.9	CP023467
**12**	*D*. *dadantii* NCPPB 3537	31.3	28	48.9	AOOL01000000
**13**	*D*. *chrysanthemi* NCPPB 516	31	27	47.6	AOOC00000000
**14**	*D*. *sp*. NCPPB 3274	36.1	31	50.2	AOOH01000000
**15**	*D*. *dadantii* 3937	32.1	26	48.1	CP002038
*	*D*. *paradisiaca* NCPPB 2511	6.8	5	51.9	AONV01000000
**G4C Group II**					
**1**	*D*. *sp*. 2B12	34.7	30	53.1	JSYG00000000
**2**	*D*. *sp*. FVG1-MFV-O17	34.7	30	53.1	RJLR00000000
**3**	*D*. *sp*. FVG10-MFV-A16	34.8	30	53.1	RJLS00000000
**4**	*D*. *zeae* EC1	34.8	30	50.8	CP006929
**5**	*D*. *zeae* DZ2Q	34.8	30	50.9	APMV00000000
**6**	*D*. *zeae* Ech586	34.8	30	51.4	CP001836
**7**	*D*. *zeae* MS1	34.9	30	51.2	APWM00000000
**8**	*D*. *zeae* NCPPB 3531	34.7	30	51.2	AOOI01000000
**9**	*D*. *zeae* ZJU1202	34.8	30	50.8	AJVN00000000
**10**	*D*. *fangzhongdai* B16	36.7	31	55.4	JXBN00000000
**11**	*D*. *fangzhongdai* S1	36.7	31	55.4	JXBO00000000
**12**	*D*. *sp*. MK7	36.7	31	55.4	AOOO01000000
**13**	*D*. *sp*. Secpp 1600	36.7	31	55.3	CP023484
**14**	*D*. *fangzhongdai* DSM 101947	37.8	32	55.2	CP025003
**15**	*D*. *fangzhongdai* M005	37.6	32	55.1	JSXD00000000
**16**	*D*. *fangzhongdai* ND14b	37.6	32	55.1	CP009460
**17**	*D*. *fangzhongdai* PA1	37.8	32	55.2	CP020872
**18**	*D*. *dadantii subsp*. *dieffenbachiae* NCPPB 2976	37.4	32	55.3	AOOG01000000
**19**	*D*. *zeae* MK19	35.6	31	51.2	AOOR01000000
**20**	*D*. *zeae* MS2	35.6	31	51.2	CP025799
**21**	*D*. *zeae* NCPPB 2538	35.6	31	51.2	AOOF01000000
**22**	*D*. *zeae* NCPPB 3532	35.6	31	51.2	AONW01000000
**23**	*D*. *solani* IPO 2222	35.9	31	54.8	CP015137
**24**	*D*. *solani* D s0432-1	35.9	31	54.8	CP017453
**25**	*D*. *solani* F012	35.9	31	54.8	PDVN00000000
**26**	*D*. *solani* GBBC 2040	35.9	31	54.8	AONX01000000
**27**	*D*. *solani* IFB0223	35.9	31	54.8	CP024710
**28**	*D*. *solani* IFB 0099	35.9	31	54.8	JXRS01000000
**29**	*D*. *solani* IFB_0158	35.9	31	54.8	PENA00000000
**30**	*D*. *solani* IFB_0221	35.9	31	54.8	PEMZ00000000
**31**	*D*. *solani* MK10	35.9	31	54.8	AOOP01000000
**32**	*D*. *solani* MK16	35.9	31	54.8	AOOQ01000000
**33**	*D*. *solani* PPO 9019	35.9	31	54.8	CP017454
**34**	*D*. *solani* PPO 9134	35.9	31	54.8	JWLT00000000
**35**	*D*. *solani* RNS 07.7.3B	35.9	31	54.8	JWLR00000000
**36**	*D*. *solani* RNS 08.23.3.1.A	35.9	31	54.8	CP016928
**37**	*D*. *chrysanthemi* NCPPB 3533	33.5	28	53.1	AOOJ00000000
**38**	*D*. *solani* RNS 05.1.2A	36.6	31	54.0	JWMJ00000000

### Identification and annotation of G4C (OAg-capsule) and OAg-LPS biosynthesis gene clusters

An NCBI blastp homology tool was used to find the OAg-capsule and OAg-LPS gene cluster within the genus *Dickeya*. Briefly, we used *wza*, *wzb*, *wzc* and *rfbB*, *rfbA*, *rfbC* protein sequences of *Dickeya solani* type strain IPO 2222T (NCBI Accession no. CP015137.1) for blastp query (S1 File) to identify OAg-capsule cluster in the non-redundant protein sequence (nr) database of NCBI in genus *Dickeya* (taxid:204037). Similarly, for identification of OAg-LPS biosynthesis gene clusters, we used *wzm* and *wzx* protein sequence of *Pseudomonas aeroginosa* PAO1 (NCBI Accession AAG08836.1 and AAG06541.1 respectively) (S1 File). We used blastp, E-value cut-off of 2e-06 for the study. We could identify these clusters in 16 out of 54 *Dickeya* strains (Table 1), for which a complete annotated genome was available. For further investigation, we downloaded genomes of 54 *Dickeya* strains (Table 1) from the NCBI genome database as the nucleotide (.fna) file and created the nucleotide blast database using standalone NCBI blast+ facility [28]. We took conserved flanking genes of OAg-capsule, OAg-LPS gene cluster, and other gene clusters described in the result section and performed tblastn and blastn search against the nucleotide blast database. The results of tblatn and blastn helped to identify the full gene cluster with precise coordinates of nucleotides. We extracted the sequences of these clusters from their genomes and used the RAST annotation server (http://rast.nmpdr.org/) to annotate them [29]. After annotation, we manually verified each Open Reading Frame (ORF) for their homology to genes from the NCBI database using blastp and further manually curated them for analysis. We generated the annotated OAg-capsule and OAg-LPS clusters using EasyFig software [30]⁠ and enhanced their appearance using Adobe illustrator CS6 software.

### Taxonomic and phylogenetic analysis of *Dickeya* strains

Phylogenetic analysis based on the complete *rpoB* gene was performed by clustalW alignment and MEGA X software [26, 31]. Similarly, taxonomic classification based on the complete *rpoB* gene was obtained by NCBI BLASTn with a ≤ 97.7% sequence identity cut-off was used as a species selection criterion [26]. The entire *rpoB* gene sequence identity was determined by the Unipro-Ugene toolkit [32]. Whole genome-based species criteria like Average Nucleotide Identity (ANI) and digital DNA-DNA hybridization (dDDH) of ≥ 94.4% and ≥ 70% were used respectively based on the standard recommendations [33–35]. Whole genome-based ANI were calculated using Jspecies software, and ANIb values were obtained [35]. Similarly, dDDH recommended values were obtained using the Genome-to-Genome Distance Calculator web server (https://ggdc.dsmz.de/) [36]. All the sequences for the identified clusters are made available at figshare repository with following link “https://figshare.com/s/91c9625e09e09bda5df3”.

## Results

### Identification of polysaccharide biosynthesis clusters in *Dickeya*

In the revolutionary era of genomics, we have access to tremendous genomic sequence data from the public database, such as NCBI (NCBI Resource Coordinators, 2016). We retrieved 73 complete and draft whole-genome sequences of members of the genus *Dickeya* from NCBI, as of March 5^th^, 2019. We further limited our selection to 54 strains, in which we were able to obtain full sequences of the group 4 capsule (G4C) coding region from whole-genome sequences (Table 1).

We identified conserved polysaccharide biosynthesis gene clusters in *Dickeya* and successfully characterized five such conserved genomic regions, including putative O-antigen clusters, with exceptions to certain species/strains. In this study, we further provide comprehensive comparisons of diverse group 4 capsule (G4C)/OAg-capsules, putative O-antigen lipopolysaccharide (OAg-LPS) coding clusters, enterobacterial common antigen (ECA) gene clusters, core-lipopolysaccharide biosynthesis clusters, and an additional alternative O-antigen biosynthesis lipopolysaccharide region in these strains.

### Identification of Group 4 Capsule (G4C) or OAg-capsule coding region in *Dickeya*

**Fig 1:**
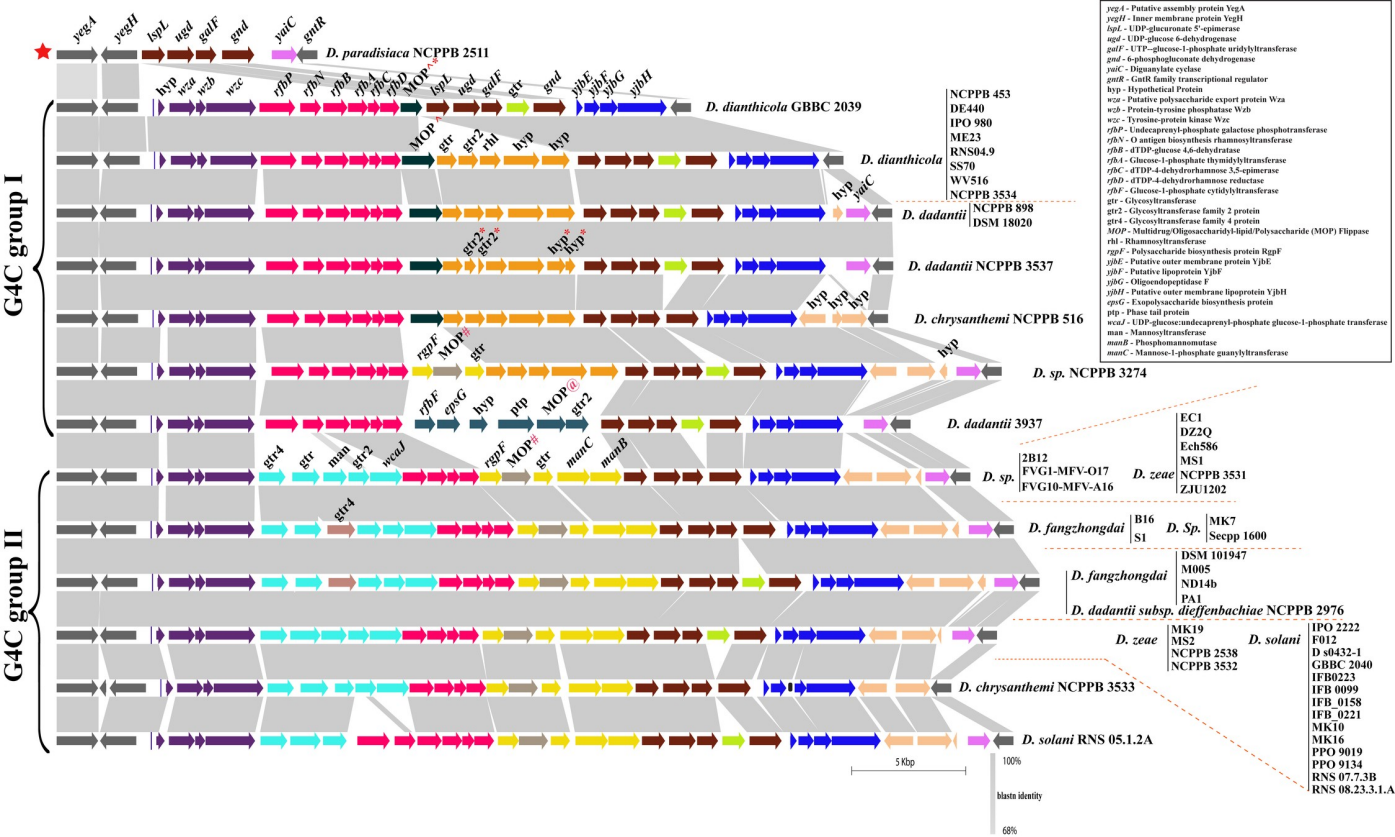
Group 4 capsule or O-antigen capsule coding biosynthesis gene clusters of *Dickeya*. Conserved flanking genes are shown in grey on both ends. Gene abbreviations are mentioned in the right corner box. JUMPstart sequence homology was marked with blue bar after initial flanking gene *yegH*. Identical sequence homology for ORFs or clusters of ORFs are shown by using the same color. Gene names and abbreviations are shown in the figure. Blastn identity is shown by a grey color scale among the clusters. Blastn identity is shown by a grey color scale among the clusters. O-antigen diversity is grouped into two major G4C groups, group I and group II, however an exception to this is *D*. *paradisiaca* NCPPB 2511 marked as red star at the beginning of the figure Respective strain names are mentioned for each cluster at the right end with red dotted boundaries. Different strains of the same species are listed as a vertical bar. @, #, and ^ denote different homologs of the corresponding gene. * denotes a pseudogene. All gene clusters are drawn to scale.

The group 4 capsule (G4C) polysaccharides are frequently identical to the cognate lipopolysaccharide O side chain. Therefore, it is often referred to as the O-antigen capsule (OAg-capsule) in *E*. *coli* [37]. Biosynthesis of various polysaccharides, including O-antigen, are often linked to the 39 bp conserved JUMPstart (for “just upstream of many polysaccharides starts”) element present at the beginning of these clusters [38]. Here, we found conserved JUMPstart sequence homology at the beginning of the cluster (marked as the blue bar, Fig 1) in the genus *Dickeya*, except for strain *Dickeya paradisiaca* NCPPB 2511. Sequence homology and alignment to the 39 bp JUMPstart sequence in *Dickeya* is shown in S1 Fig. These G4C cluster genes are flanked by membrane protein YegA and transport protein YegH (*yegA-yegH*) on the left and transcriptional regulator GntR (*gntR*) on the right as shown in grey color (Fig 1). All 54 studied genomes of *Dickeya* strains contain this conserved locus in their genomes.

Based on their sequence identities and variation, we studied these clusters individually and grouped them as represented in Fig 1. There are two major types of G4C cluster groups, i.e., G4C group I and G4C group II as marked in Fig 1 (Table 1). These groups are distributed among different species of *Dickeya* and exhibit diversity. The average GC content of G4C clusters is significantly different compared to their average genomic GC content (Table 1). This feature indicates the possible role of HGT events to shape their G4C cluster organization. These G4C clusters also vary in size as well as numbers of Open Reading Frames (ORFs), as listed in Table 1.

The beginning of both group I and group II G4C clusters contain three ORFs including *wza*, *wzb*, and *wzc* genes that are respectively similar to *yccZ-etp-etk* genes of the *cps/wca* operon responsible for the production of colanic acid (CA)-EPS synthesis (Fig 1, violet color) [37, 39]. Another *yjbEFGH* operon (marked in blue in Fig 1) was also consistently present in both the G4C group strains and probably has a role in exopolysaccharide biosynthesis [37, 40]. This operon is also paralogous to the *ymcDCBA* operon known for O-antigen biosynthesis in *E*. *coli* [37, 40]. However, the role of the *yjbEFGH* operon is uncertain in *Dickeya* but is speculated to be part of O-antigen capsule biosynthesis.

### Structure, variation, and diversity of G4C (O-antigen capsule) group I coding region in *Dickeya*

Fifteen *Dickeya* strains contain group I G4C cluster (Table 1). The G4C group I cluster is characterized by the presence of both a set of six genes, i.e., *rfbP*, *rfbN*, *rfbB*, *rfbA*, *rfbC*, *rfbD* and a set of five genes i.e., *gtr*, *gtr2*, *rhl*, and two *hyp*s clustered within the conserved flanking genes (Fig 1). Four genes from these clusters, *rfbB*, *rfbA*, *rfbC*, and *rfbD*, are known to be involved in dTDP-rhamnose biosynthesis *rfbBACD* operon, which is an important precursor for the biosynthesis of various cell-wall polysaccharides, EPS and LPS [41, 42]. This *rfbBACD* operon is present in all G4C group I and II strains, likely coding for O-antigen (Fig 1). However, five genes, encoding for two glycosyltransferases, a rhamnosyltransferase, and two hypothetical proteins, are uniquely present in G4C group I members except for *D*. *dianthicola* GBBC 2039 and *D*. *dadantii* 3937 (shown in orange color, Fig 1). In *D*. *dadantii* NCPPB 3537, glycosyltransferase and the hypothetical proteins from the five gene clusters are present as pseudogenes (marked with *, in Fig 1). Furthermore, a multidrug/oligosaccharidyl-lipid/polysaccharide (MOP) flippase coding ORF is present in all the group I members, just after the *rfbD* gene. In *D*. *sp*. NCPPB 3274 and *D*. *dadantii* 3937, different homologs of MOP flippase are present as a cluster of three and six genes, respectively (Fig 1). The cluster of three genes in *D*. *sp*. NCPPB 3274 contains *rgpF*, MOP^**#**^ flippase, and a glycosyltransferase (Fig 1). Similarly, the six genes with distinct MOP^**@**^ flippase in *D*. *dadantii* 3937 encodes for *rgbF*, *epsG*, hypothetical protein (hyp), phage-tail protein (ptp), MOP^**@**^ flippase (MOP^**@**^) and glycosyltransferase family 2 protein (*gtr2*).

Interestingly, we found that *D*. *paradisiaca* NCPPB 2511 contains smallest G4C cluster of 6.8 Kbp (Kilobasepair) and consists of only five ORFs which encode for *lspL*, *ugd*, *galF*, *gnd*, and *yaiC* genes. Four out of these five genes namely *lspL*, *ugd*, *galF*, and *gnd*, are present in both of the G4C clusters group I and II, as shown in brown color in Fig 1 and Table 1. Our phylogenetic distribution study (Fig 4) also indicates *D*. *paradisiaca* NCPPB 2511 to be evolutionary ancestral strain. Owing to presence of its genes in both the G4C group I and II cluster we have denoted as star marked individual cluster (Fig 1).

In this study, we found that *D*. *dianthicola* strains and the major *D*. *dadantii* species exclusively contains G4C group I clusters. However, one of *D*. *chrysanthemi* strain, *D*. *chrysanthemi* NCPPB 516 is also part of the G4C group I cluster. The most extended LPS group I cluster of 36.1 Kb with 31 ORFs was present in strain *D*. *sp*. NCPPB 3274, (Table 1).

### Structure and variation of G4C (O-antigen capsule) group II coding region in *Dickeya*

A total of 38 out of 54 strains contain G4C group II cluster homology (Table 1 and Fig 1). Our findings suggest that the genus *Dickeya*, *D*. *fangzhongdai* and *D*. *solani* species exclusively belong to G4C group II clusters. The smallest G4C group II cluster is 33.5 Kb, with 28 predicted ORFs in strain *D*. *chrysanthemi* NCPPB 3533. *D*. *chrysanthemi* is present in both G4C groups I and II with its single representative strains in each of the group. The largest group II G4C cluster is represented by *D*. *fangzhongdai* species and *D*. *dadantii subsp*. *Dieffenbachiae* NCPPB 2976 with a length of 37.8 Kb encoding 32 ORFs. G4C group II clusters appear differently from the G4C group I members in that they have two distinct five gene clusters shown in the sky-blue and yellow-colored region in Fig 1. The first five gene cluster consists of *gtr4*, *gtr*, *man*, *gtr2* and *wcaJ* genes, while the second five gene cluster is represented by *rgpF*, *MOP*^**#**^, gtr, *manC*, and *manB* respectively (Fig 1). A slight variation of an additional *gtr4* gene (shown in light brown color) in the first five gene cluster was present in species *D*. *fangzhongdai* and a few other strains (Fig 1).

The *MOP*^**#**^ flippase gene, colored in olive, is present in all members with G4C group II clusters and in one G4C group I member, *D*. *sp*. NCPPB 3274. The *MOP* genes were present in three different gene sequences, and out of these, *MOP*^^^ is characteristic of G4C group I while *MOP*^**#**^ is present in G4C group II containing *Dickeya* species except for *D*. *sp*. NCPPB 3274. In *D*. *sp*. NCPPB 3274, *MOP*^#^ is present with the arrangement of three genes (*rgpF*, *MOP*^***#***^, *gtr*). The third *MOP*^**@**^ is present in *D*. *dadantii* 3937 of G4C group I. An additional glycosyltransferase (*gtr*), shown in bright green, was also present in different G4C group I and II cluster strains (Fig 1). *D*. *solani* RNS 05.1.2A contains *rfbP* and *rfbN* genes followed by the *rfbBACD* operon, similar to the G4C cluster group I members.

Despite finding distinct *MOP* flippase genes in these G4C clusters, the most prevalent O-antigen processing gene, *wzx* (flippase), *wzy* (polymerase), or *wzm*/*wzt* transport system (ABC transporters) [43]⁠ are not present within this locus.

### Identification of putative O-antigen lipopolysaccharide (OAg-LPS) biosynthesis cluster

**Fig 2:**
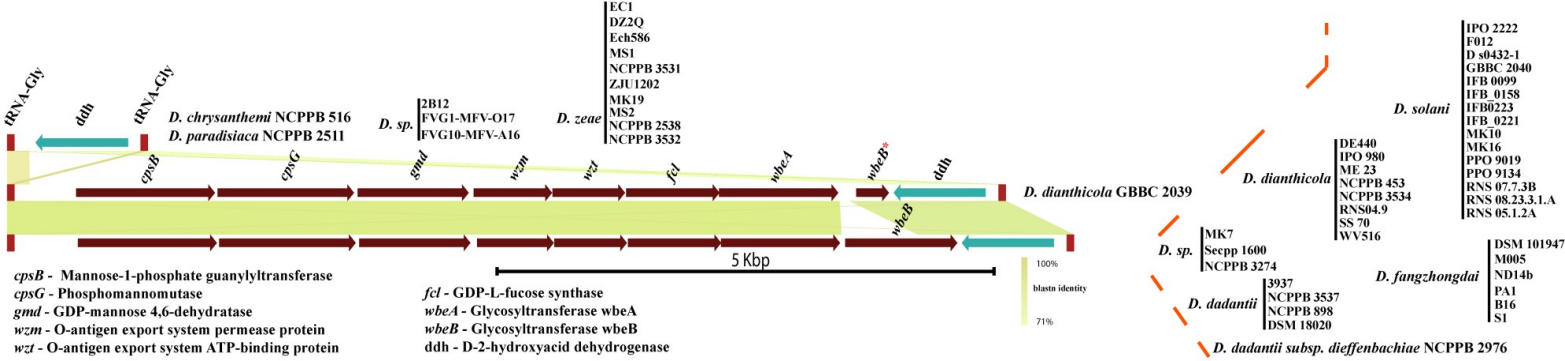
Putative O-antigen lipopolysaccharide biosynthesis gene cluster in genus *Dickeya*. Gene abbreviations are mentioned in the bottom-left corner. Identical colors show ORFs or clusters of ORFs with identical sequence homology. Gene names and abbreviations are shown in the figure. Blastn identity is shown with a green color scale among the clusters. Respective strain names are mentioned for each cluster at the right end with red dotted boundaries. Different strains of the same species are listed as a vertical bar. * denotes a pseudogene. All gene clusters are drawn to scale.

We further investigated the presence of *wzx* and *wzm* homologs in the genomes of *Dickeya*. Our findings suggest the presence of a putative O-antigen lipopolysaccharide (OAg-LPS) biosynthesis cluster in *Dickeya*. This particular locus contains an essential O-antigen transport system (*wzm-wzt* transport genes) with a cluster of eight genes—*cpsB*, *cpsG*, *gmd*, *wzm*, *wzt*, *fcl*, *wbeA*, and *wbeB* (Fig 2). This locus is flanked by the tRNA-Gly gene on the left and a D-2-hydroxy-acid dehydrogenase (*ddh*) gene on the right, followed by another tRNA-Gly gene (Fig 2). *D*. *dianthicola* GBBC 2039 contains *wbeB* as a pseudogene in this cluster. The most critical *wzm*-*wzt* genes are part of this conserved cluster, contributing to O-antigen transport in the periplasm. This small but quintessential putative O-antigen cluster contains colanic acid biosynthesis genes *cpsB* and *cpsG*. It also contains the GDP-L-fucose biosynthesis genes *gmd* and *fcl*, which are part of the colanic acid (CA) biosynthesis cluster in *E*. *coli* [44]. This locus is conserved in all 54 strains. Interestingly, a total of 15 strains, shown in Fig 2, do not contain LPS coding genes and end abruptly with only the *ddh* flanking ORF between two tRNA-Gly genes (Fig 2). This arrangement also occurs in all *D*. *zeae* species members.

Since the O-antigen transport system is essential for all the species of *Dickeya*, we searched for it across the genomes of the remaining strains and did not find this putative O-antigen cluster at this locus. Instead, we found these transport genes along with LPS biosynthetic cluster at different locus, as described in the next section.

### Complementary putative O-antigen lipopolysaccharide (OAg-LPS) cluster identification and characterization in *Dickeya*

**Fig 3:**
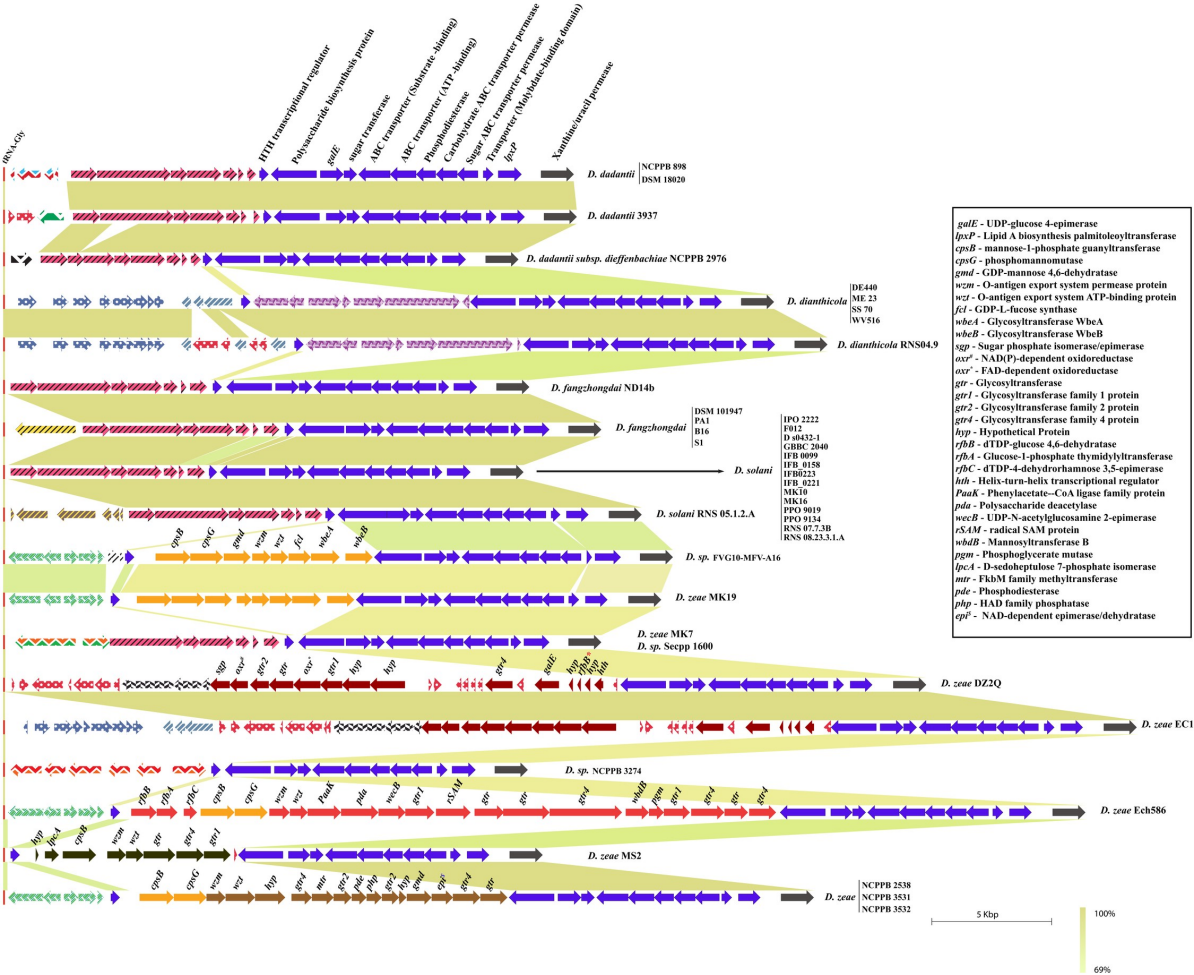
Complementary alternative O-antigen biosynthesis gene cluster in *Dickeya*. LPS biosynthesis genes are shown with solid color fill. However, non-LPS coding genes are marked with different color patterns based on their gene annotations. Identical gene homology clusters are shown with identical color or color patterns in this figure. Gene names and abbreviations are shown in the figure. * denotes a pseudogene. All clusters are drawn to scale with the blastn identity color scale.

The alternative putative O-antigen lipopolysaccharide (OAg-LPS) locus is found to be in proximity of genes involved in the lipid A biosynthesis gene, *lpxP*. Moreover, this locus is flanked by a tRNA-Gly gene on the left and xanthine/uracil permease gene on the right (Fig 3). A cluster of ten ORFs, including the *lpxP* gene on the right end of this locus, is related to polysaccharide biosynthesis and marked with a navy blue color. The annotation of these 10 ORFs reveal a polysaccharide biosynthesis protein, *galE*, sugar transferase, ABC transporters (both substrate binding and ATP-binding), phosphodiesterase, carbohydrate ABC transporter permease, molybdate binding transporter protein, and *lpxP* gene. The role of this cluster of genes is not yet known and requires further experimental evidence.

However, the missing putative OAg-LPS cluster genes, as described in the previous section, are mostly present in the remaining 15 strains. As shown in Fig 3, ORFs marked with different color patterns do not show any relation to LPS biosynthesis genes according to their *in silico* functional annotation. ORFs in different color patterns are also marked based on identical nucleotide homology and clustering.

Out of these 15 *Dickeya* strains which lack putative OAg-LPS cluster, we identified a complete alternative OAg-LPS cluster in nine of them (Fig 3). *D*. *sp*. FVG10-MFV-A16 and *D*. *zeae* MK19 contain an identical arrangement of eight ORFs (*cpsB*, *cpsG*, *gmd*, *wzm*, *wzt*, *fcl*, *wbeA*, and *wbeB*) in a putative OAg-LPS cluster, just upstream of a polysaccharide biosynthesis protein (shown in orange color, Fig 3).

In *D*. *zeae* DZ2Q and *D*. *zeae* EC1, eight gene clusters (*sgp*, *oxr*^#^, *gtr2*, *gtr*, oxr^*^, *gtr1*, two *hyp*s) are related to LPS biosynthesis, as shown in brown (Fig 3). Few other genes like *gtr4*, *galE*, rfbB*, two *hyp*, and *hth* related to LPS biosynthesis are also present and marked in brown for both the strains. The rfbB* gene is present as a pseudogene in both strains, *D*. *zeae* DZ2Q and *D*. *zeae* EC1. These strains lack *wzm*-*wzt* transporters at this locus. This ambiguity remains open for further investigation and discussion.

*D*. *zeae* NCPPB 2538, *D*. *zeae* NCPPB 3531, and *D*. *zeae* NCPPB 3532 have identical cluster arrangement, which contains 16 LPS biosynthesis related ORFs in addition to a 10 ORFs cluster at the end of this locus. *D*. *zeae* Ech586 and *D*. *zeae* MS2 strains contain entirely different clusters containing distinct *wzm-wzt* homologs. Individual ORFs and clusters of ORFs annotated with abbreviations and specific color are shown in Fig 3. In addition to the G4C group I cluster shown in Fig 1, *D*.*zeae* Ech586 contains a duplicate set of *rfbB*, *rfbA*, and *rfbC* at an alternative locus (Fig 3).

### Phylogenetic distribution of O-antigen capsule (G4C) coding region across the genus *Dickeya*

**Fig 4:**
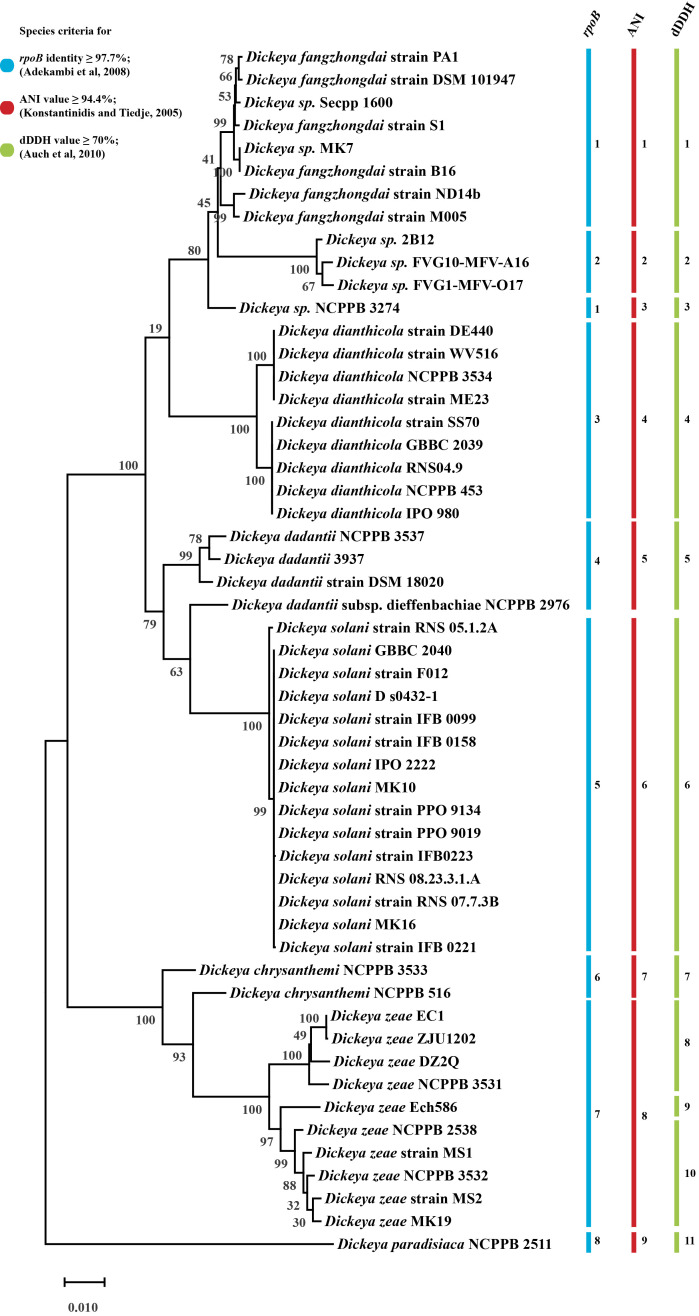
Maximum-likelihood phylogenetic tree of *Dickeya* based on the complete *rpoB* sequence and its species delineation. The phylogenetic tree was obtained by clustalW alignment with a complete deletion method and 500 bootstrap values. Individual color bars and respective species group numbers have been assigned to different species criteria of *rpoB*, ANI, and dDDH.

Phylogenetic species groups were determined with whole genome-based species criteria of Average Nucleotide Identity (ANI) and digital DNA-DNA hybridization (dDDH) methods. Our study encompassing 54 *Dickeya* strains suggests the presence of nine species groups, based on ANI species criteria (≥ 94.4% identity) and 11 species groups, based on dDDH criteria (≥ 70% identity) (S2 Fig). Members of *D*. *zeae* are divided into three distinct species groups based on dDDH classification. We also used RNA polymerase beta subunit (*rpoB*) gene, found as single copy gene in bacterial genome and relatively immune to HGT, as a phylogenetic marker and a species delineation cut-off of ≥ 97.7% sequence identity, as interpreted in Fig 4 [26, 27]⁠ (53 strains are used for this analysis, data available in S1 Table). It shows eight distinct *rpoB* species grouping of 53 *Dickeya* strains and has similarity to the findings with ANI, except for strain *D*. *sp*. NCPPB 3274 (Fig 4). We compared the different G4C clusters present with each strain and their phylogenetic distance with each other (S3 Fig).

In our study of the variation of G4C (O-antigen capsule) group I and II coding region in *Dickeya*, we found that the G4C group I cluster is predominantly present in *D*. *dianthicola*, and *D*. *dadantii* strains and the G4C group II cluster is present in *D*. *fangzhongadi*, *D*. *zeae*, and *D*. *solani*, while *D*. *sp*. and *D*. *chrysanthemi* are distributed in both G4C group I and II clusters. These observations are not consistent with variation of putative O-antigen LPS biosynthesis cluster, and an alternative O-antigen LPS biosynthesis clusters in those species which may contribute to more variation of O-antigen of individual species.

### Enterobacterial Common Antigen (ECA) biosynthesis cluster in *Dickeya*

**Fig 5:**
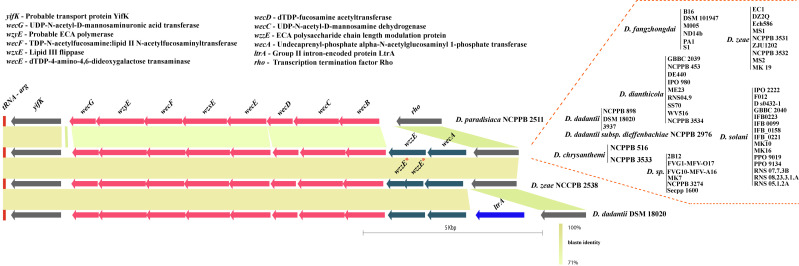
Enterobacterial Common Antigen (ECA) biosynthesis clusters in *Dickeya*. * denotes a pseudogene. Gene abbreviations are mentioned at the top of the figure. All clusters are drawn to scale.

The ECA biosynthesis cluster, or *wec* cluster, in *Dickeya* is flanked by conserved genes *yifK* on the left and *rho* on the right end. Additionally, a tRNA-Arg gene is present just before the *yifK* flanking gene. This locus is highly conserved with its constituent genomic organization. Fiftyone out of 54 studied strains contain an identical set of ten ORFs, namely *wecG*, *wzyE*, *wecF*, *wzxE*, *wecE*, *wecD*, *wecC*, *wecB*, *wzzE*, and *wecA*, between these flanking genes (Fig 5). However, *D*. *paradisiaca* NCPPB 2511 lacks *wzzE* and *wecA* at this locus. *D*. *zeae* NCPPB 2538 contains *wzzE* as a pseudogene in their cluster (Fig 5). *D*. *dadantii* DSM 18020 contains an additional ORF coding for the *ltrA* gene in this locus.

### Core lipopolysaccharide biosynthesis *waa* cluster diversity in *Dickeya*

**Fig 6:**
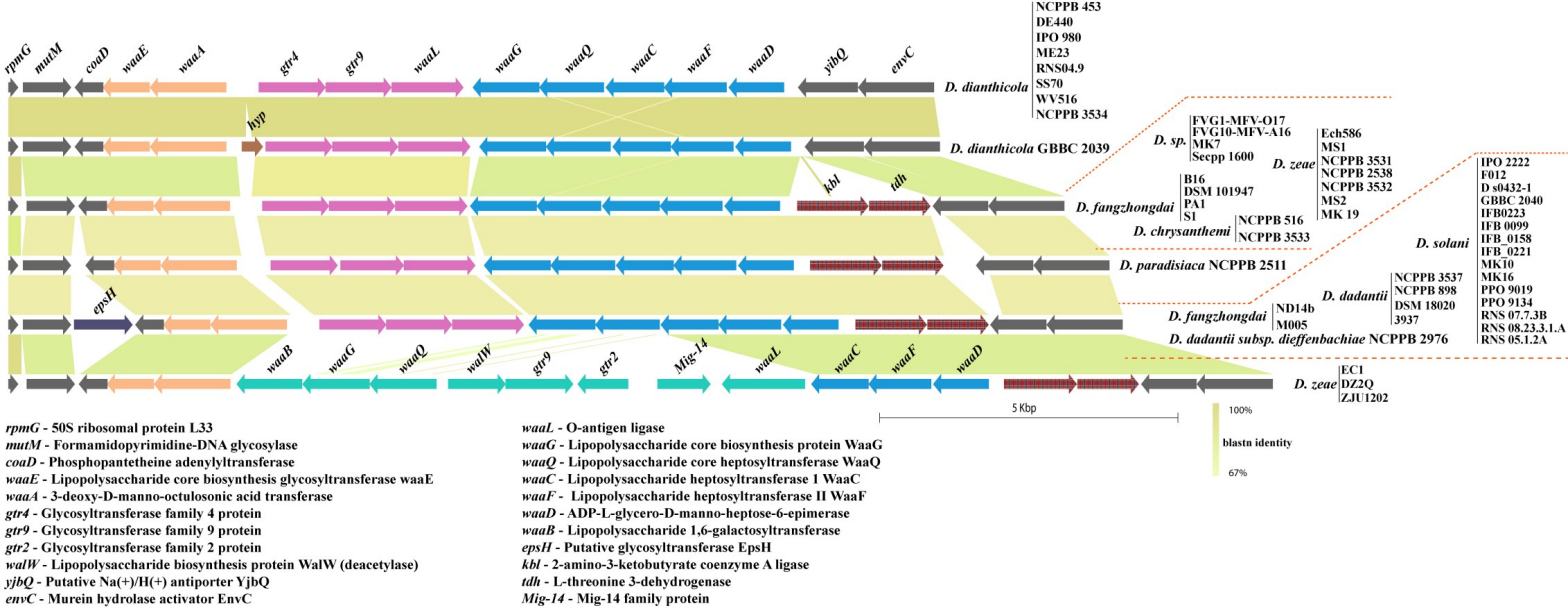
Core lipopolysaccharide biosynthesis (*waa*) cluster diversity in *Dickeya*. Identical sequence homology for ORFs or clusters of ORFs are shown with matching color. Gene abbreviations are mentioned at the bottom of the figure. All clusters are drawn to scale.

The *waa* gene cluster involved in core lipopolysaccharide biosynthesis in *Klebsiella pneumoniae* is present, between the *coaD* and *kbl* flanking genes [45]. A substantial similarity of this conserved locus is found in several species of *Dickeya*. However, in case of *D*. *dianthicola*, this locus is flanked by *coaD* and *yjbQ*. The *yjbQ* gene is the second downstream gene to *kbl* in other *Dickeya* strains (Fig 6). The majority of *Dickeya* members contain an array of 10 genes, i.e., *waaE*, *waaA*, *gtr4*, *gtr9*, *waaL*, *waaG*, *waaQ*, *waaC*, *waaF*, and *waaD* that constitutes the *waa* gene cluster (Fig 6). One additional *epsH* gene is also present before the *coaD* flanking gene in several strains of *D*. *solani*, *D*. *dadantii*, and *D*. *fangzhongdai* (Fig 6). *D*. *zeae* EC1, *D*. *zeae* DZ2Q, and *D*. *zeae* ZJU1202 contain different homologs of *waaB*, *waaG*, *waaQ*, *waaL*, and other genes *walW*, *gtr9*, *gtr2*, and a *Mig-14*, shown in green (Fig 6).

## Discussion

The genus *Dickeya* is a significant threat to herbaceous plants and vegetables. In Gram-negative bacteria, complex polysaccharides form an essential component of the bacterial outer surface. The surface polysaccharides enable bacteria to colonize the host and cause disease by evading plant immunity [46]. ⁠It is estimated that more than one capsule system is present in 40% of the total analyzed bacterial lineages [47]. The present study describes the genetic relatedness of the complete lipopolysaccharide O-antigen and G4C biosynthesis systems in eight distinct species of *Dickeya* encompassing 54 strains.

LPSs are recognized as one of the crucial pathogen-associated molecular patterns (PAMPs) and potent elicitors of PAMP triggered immunity (PTI) [48]. Long-chain O-antigen enables *Xylella fastidiosa* to delay host innate immune recognition and allows for effective establishment into the host [49]. The localized induced response is a common phenomenon and is observed in pepper leaves after treatment with different LPS components isolated from *Xanthomonas campestris* [50]. These responses include the production of phenolic compounds, alteration in the expression of pathogenesis related (PR) proteins, and prevention of HR responses caused by avirulent bacteria [51, 52]. The effect of different components of LPS of genus *Dickeya* on plant responses is not yet completely understood. Additionally, the revelation of LPS and its genomic counterparts as an important virulence factor remain mostly unidentified in the genus *Dickeya*.

The O-antigen capsule biosynthesis clusters in *Dickeya* are categorized into two different G4C groups as they are distinct from each other in their genetic makeup. This suggests a parallel co-evolution of the two G4C groups through genetic recombination among these strains or mutations in their genomes. The uniform structure of O-polysaccharides (a homopolymer of 6-deoxy-d-altrose: →2)-β-d-6dAlt*p*-(1→) isolated from *Dickeya solani* strains from different geographic regions has been reported [53]. This observation is in agreement with our finding of a highly clonal arrangement of a putative G4C group II cluster and O-antigen LPS in *D*. *solani*, as shown in Figs 1 and 2. Other polysaccharide biosynthesis clusters are also consistently conserved for *D*. *solani* (Figs 3, 5, and 6). However, the novel differences between G4C groups I and II and other polysaccharide biosynthesis clusters might produce distinct O-antigen capsules but remain to be experimentally validated for the genus *Dickeya*. Enteropathogenic *Escherichia coli* is resistant to human α-defensin 5 and is linked to the additive effects of both G4C and lipopolysaccharide O-antigen [54]. Subsequently, both G4C and O-antigen LPS may contribute to virulence, pathogenicity, and better survival of *Dickeya* in plants, therefore altering host-pathogen interactions.

Many enterobacteria produce colanic acid (CA) or M-antigen as an exopolysaccharide (a negatively charged polymer of glucose, galactose, fucose, and glucuronic acid) [55]. However, LPSs act as a potent stimulator of innate or natural immunity in diverse eukaryotic species ranging from insects to humans [56]. This study also suggests that the modification of lipopolysaccharides with colanic acid might be possible in *Dickeya*, as LPS and colanic acid biosynthesis genes are related in origin and intermingled at these clusters. This combination of colanic acid, LPS core biosynthesis cluster, and *waaL* are responsible for the presence of M-antigen, which is a modified form of O-antigen in *E*. *coli* [55]. However, while this may be possible in *Dickeya*, it would be presumptive to conclude that such M-antigen is present and contributes to their diversity of surface molecules.

A recent study shows that genetic exchanges in bacteria encoding capsules are frequent and lead to changes in genome dynamics [46, 57]. Variation at the LPS biosynthesis cluster in phytopathogenic Gram-negative bacteria, *Xanthomonas oryzae* pv. *oryzae* (cause of bacterial blight of rice) is driven by multiple HGT events [58]. Continuing this observation of the O-antigen capsule/G4C coding genomic region in *Dickeya* indicates multiple HGT events and are responsible for the structure of the current complex polysaccharide biosynthesis cluster diversity. The presence of several t-RNA coding genes at the sites of complex polysaccharide biosynthesis clusters in *Dickeya* suggests that tRNA genes are favorable sites for recombination/integration of these LPS/Capsule coding pathogenicity clusters. These properties of G4C capsule and LPS biosynthesis clusters, preferentially linked to tRNA sites, could be prevalent in phytopathogenic bacteria and needs further investigation with a more diverse group of bacterial populations. Moreover, these clusters meet the classical features of pathogenicity islands in several cases [59, 60]⁠.

ECA and core lipopolysaccharide biosynthesis clusters (*waa*) are highly predictable and consistent in their genetic organization with less variation. However, the *Dickeya* genus, being a member of *Enterobacteriaceae*, is expected to have ECA and *waa* gene clusters with less or no variation (Figs 5 and 6). A recent transcriptomics and comparative genomics study using 100 genomes of *Pectobacterium* and *Dickeya spp*. identified the structural organization of G4C, *wec*, and *waa* gene clusters in *Pectobacterium carotovorum* subsp. *brasiliense* strain PBR 1692 [61]. Their experimental findings suggest further investigation is needed in these species, including *Dickeya*. We present a thorough comparative analysis of LPS biosynthesis and O-antigen capsule biosynthesis clusters present in *Dickeya*. In *D*. *zeae*, the heterogeneity of O-antigen capsule and *waa* gene clusters might be one of the several factors affecting their virulence differentially, as reported by Ming Hu and co-workers [62]. Apart from pathogens, the capsular proteins found in many non-pathogens may increase their ability to survive in constantly changing environmental conditions suggesting its role in niche specialization [63]. In turn, it requires extensive experimental evidence to ascertain the role of the different genetic organization of the O-antigen capsule coding regions to the differences observed in host-pathogen interactions. Overall, this study provides a better insight into how the genetic variations and organization of polysaccharide biosynthetic clusters may lead to changes in bacterial surface structure and therefore pathogenicity of *Dickeya*. The functional study of these genetic variations will lead to better understanding of their impact on pathogenicity.

## Supporting information

S1 Fig 39bp JUMPstart sequence homology to G4C cluster in *Dickeya*. (TIF)

S2 Fig Whole-genome ANI and dDDH value matrix of selected 54 strains of *Dickeya* with respective species cut-off criteria. Whole genome ANI species cut-off of 94.4% and above is marked with red color boxes while dDDH species cut-off of 70% and above were marked with green color boxes in this figure.
(TIF)

S3 Fig Diversity of G4C/O-antigen capsule gene clusters of *Dickeya* in relation to their phylogenetic classification based on *rpoB*, ANI, and dDDH species criteria. G4C coding clusters of 54 *Dickeya* strains were aligned to their phylogenetic tree based on *rpoB* and also classified with different color bar of ANI and dDDH. Clusters are drawn to scale and *rpoB*, ANI and dDDH species cut-off is mentioned with dot colored legends.
(TIF)

S1 Table Complete *rpoB* sequence identity (%) matrix for 53 strains of genus *Dickeya*. Sequence identity matrix with species cut-off of 97.7% or above identity is marked in blue color for the specified strains.
(XLSX)

S1 File BlastP query sequence of the studied clusters. (DOCX)
