# Supplementary figures and images for: Genomic diversity and organization of complex polysaccharide biosynthesis clusters in the genus Dickeya
Source: PLoS One. 2021 Feb 11;16(2):e0245727. doi: 10.1371/journal.pone.0245727 (PMC7877592; doi:10.1371/journal.pone.0245727)

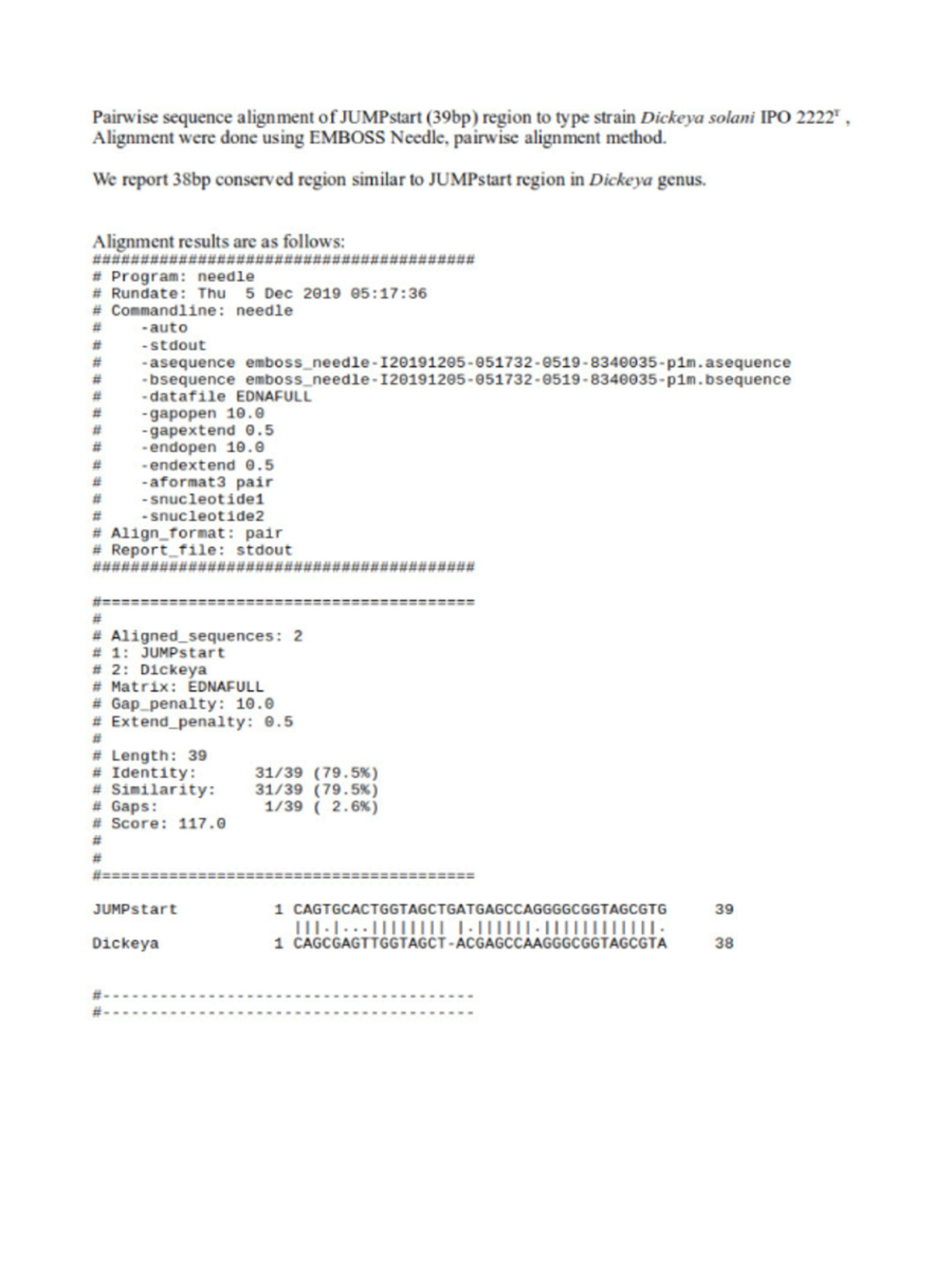

Supplement: S1 Fig — (TIF) [file pone.0245727.s001.tif]

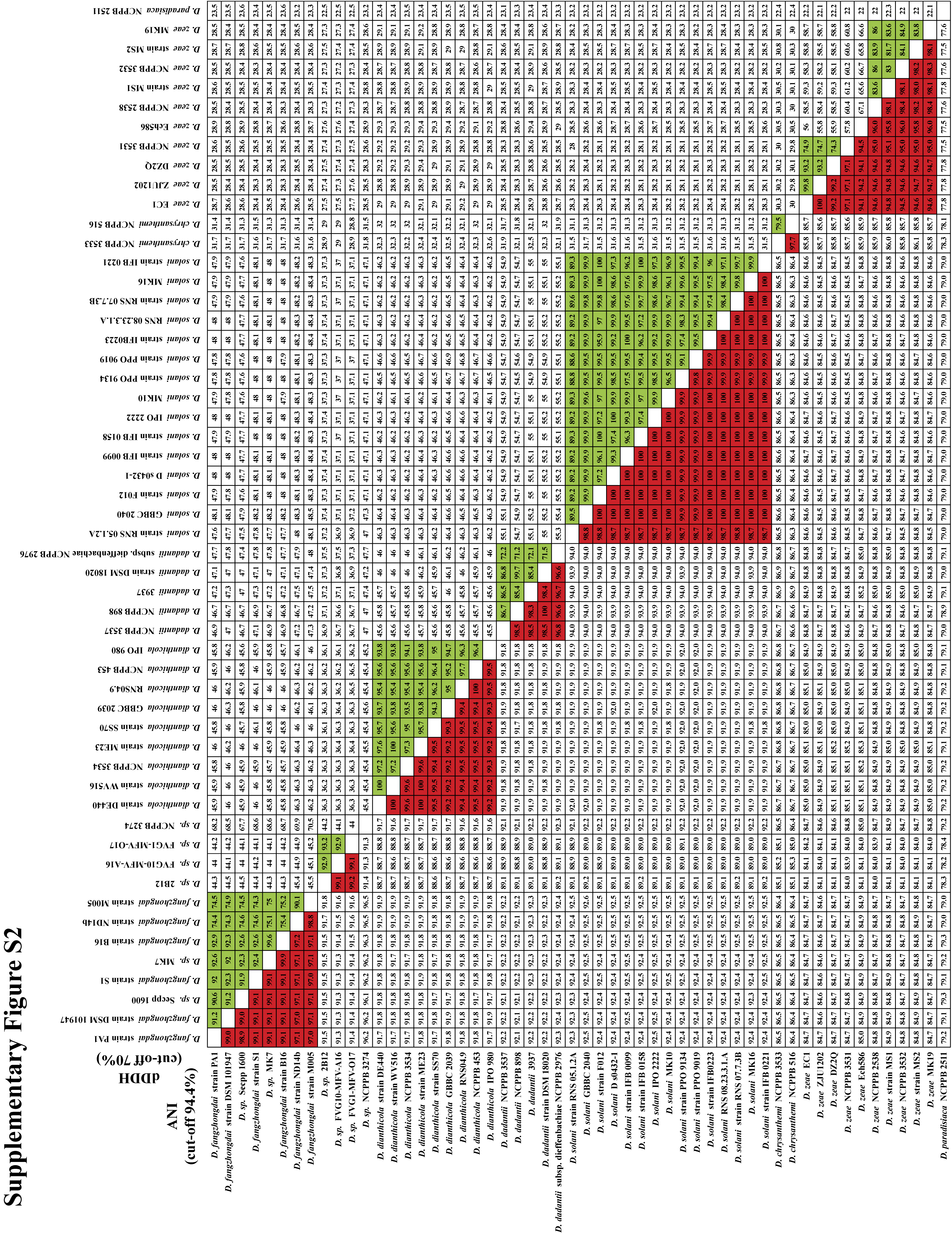

Supplement: S2 Fig — Whole genome ANI species cut-off of 94.4% and above is marked with red color boxes while dDDH species cut-off of 70% and above were marked with green color boxes in this figure. (TIF) [file pone.0245727.s002.tif]

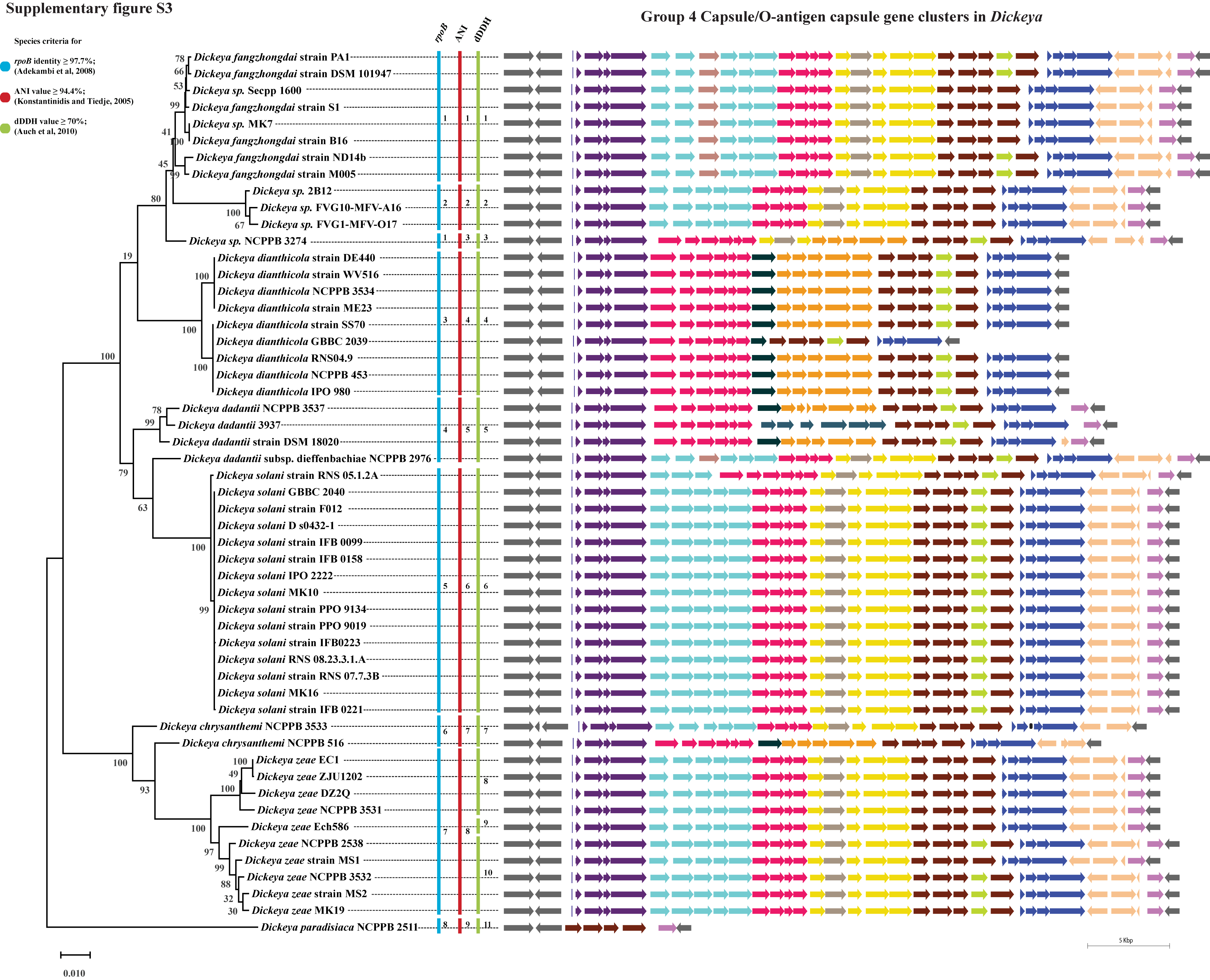

Supplement: S3 Fig — G4C coding clusters of 54 Dickeya strains were aligned to their phylogenetic tree based on rpoB and also classified with different color bar of ANI and dDDH. Clusters are drawn to scale and rpoB, ANI and dDDH species cut-off is mentioned with dot colored legends. (TIF) [file pone.0245727.s003.tif]
