# Supplementary material for: Genomic diversity and organization of complex polysaccharide biosynthesis clusters in the genus Dickeya
Source: PLoS One. 2021 Feb 11;16(2):e0245727. doi: 10.1371/journal.pone.0245727 (PMC7877592; doi:10.1371/journal.pone.0245727)
Supplement: S1 File — (DOCX) [file pone.0245727.s005.docx]

BlastP query sequence for Fig 1.

>ANE76638.1 polysaccharide export protein Wza [*Dickeya solani* IPO 2222]

MLKPTLKVIPLMLSATFLAGCTLVPGTHLSTSGKEVVEQPDDDFNINKLVNIYPVTPLLIEKIRLRPAIA

QNNPSLDAELKRYEYRIGIGDVLMVTVWDHPELTTPAGTYRTAADTGNWVHSDGTIFYPYIGKIRVVDKT

LTEVRDEIMRRLAQYIESPQVEVSIAAFRSQKAYVTGEVVRSGQQPISNVPLTILDAINNAGGLSEFADW

RNLVLTHNGKDERISLQALMQNGDLSQNRLLYPGDILFVPRNDDLKVFVMGEVNKQSTLKMDRSGMTLTE

ALGSAEGINQMVADATGVFVIRPLRGTQGPKLANIYQLNTKDATALVMGSEFPLQPYDVVYVTSAPIARW

NRLISQLVPTISGFNDLSEGSLRVRTWP

>ANE76639.1 protein tyrosine phosphatase [*Dickeya solani* IPO 2222]

MFNSILVICEGNICRSPTGERLLQQALPGKTVVSAGWRAMAGKPADETASLVAADHGLSLDGHVARQLTG

SLCRHYDLILVMEKAHIDAVCRFSPEVRGKTLLFGHWLNQQEITDPYRKSREAFEFVYTQLEQSARKWAQ

ALSR

>ANE76640.1 tyrosine protein kinase [*Dickeya solani* IPO 2222]

MSERIAVKTSEADKADEIDLGRLLGTVLDHRWLIISITTIFTMLGILYALLATPVYQADAMVQVEQNVSD

SLMNDISKVLPDTKPQSAPEVELLRSRMVVGKTVQDLGLDTEVEQQYFPLVGKGIARLKDEEPARIAVSR

LSVPDSWLDEKITVAIQDGGKYRFTAGKDAEFTGSIGQLESHGGFSLLISDTNAVAGTVFSVRKKNMLTA

INDILSELAVADKGKDTGVLQLTLEGSDPALVKKTLASISDNYLQQNVDRKSEEAARSLMFLKEQLPLVR

TSLETAEDKLNKYRQQKDSVDLSLEAKSVLDTIVDVESQLNQLTFHEAEISKLYTREHPAYRALLEKRGT

LEKERDNLNRRVSGMPKTQQEIVRLTRDVNVGQEVYVQLMNKQQELSINKASTVGNVRIIDQAVVQLKPV

KPRKTLVILLAAMLGGVASVGFVLLKTLLHRGIESPEQLEELGINVYASVPLSEWQQKKDREFVGKNRKR

NTRSDSLLAVGNPADLAIEAIRSLRTSLHFAMMEAKNNVLMISGASPGIGKSFVSANLGAVIAQAGQRVL

IVDCDMRKGYAHHLMDATPERGMSDILSGQIETQQALRSTAVENLFFIPRGQIPPNPSELLMHSNFTAFV

DWAVRQFDIVLLDTPPILAVTDAAIISRQAGTSLLVARFEMNTPKEVEISIRRFEQNGTPIKGVILNAVI

RRALSYYSYGYDSYQYSYGADKN

>ANE76645.1 dTDP-glucose 4,6-dehydratase [*Dickeya solani* IPO 2222]

MNILVTGGAGFIGSAVVRHIIQHTQDRVMVVDCLTYAGNLASLKEVASDPRFLFEKVNICDRAGLDRVFA

TFQPDAVMHLAAESHVDRSIDGPSAFIETNITGTYTLLEAARQYWLALPEASRAAFRFHHISTDEVYGDL

HGTDDLFTETTPYAPSSPYSASKAASDHLVRAWLRTYGLPTLVTNCSNNYGPYHFPEKLIPLMILNALEG

KPLPVYGQGNQIRDWLYVEDHARALYTVVTTGEVGETYNIGGHNERKNIEVVKTICALLDELRPEKPAGI

RHYDELITHVTDRPGHDLRYAIDASKIYRELGWKPQETFESGIRKTVEWYLANEAWWRSVKDGSYTGERL

GLTL

>ANE76646.1 glucose-1-phosphate thymidylyltransferase [*Dickeya solani* IPO 2222]

MKGIVLAGGSGTRLYPITRGVSKQLLPIYDKPMIYYPISVLMLAGIRDILIISTPDDLPAYRRLLGNGSR

FGVNLFYAEQPSPDGLAQAFLIGETFINGDQCALVLGDNIFFGQSFGKKLENVAARTEGATVFGYQVMDP

ERFGVVEFDDNNQAISLEEKPSKPKSNWAVTGLYFYDRHVVEMAKQVKPSARGELEITTLNEMYLQQGNL

NVEVLGRGFAWLDTGTHDSLLEASQFISTIEKRQGFKVACLEEIAFRKGWLTREQVADEARYLGKTHYGQ

YLAQLLTGM

>ANE76647.1 dTDP-4-dehydrorhamnose 3,5-epimerase [*Dickeya solani* IPO 2222]

MQVHDTAIQGVKIIQPKVFGDARGFFLETFEKNRYQEMLDIDLDFVQDNHSRSAKGVLRGLHFQTSNPQG

KLVRVVRGEVFDVVVDIRPDSPTYGRWEGVVLSEENKTQFWIPPGLAHGFVVLSELADFEYKCTDYYNPA

HEGCLLWNDADIGVDWPIASPLLSDKDQKGKAFKELWA

BlastP query sequence for Fig 2.

>AAG08836.1 membrane subunit of A-band LPS efflux transporter [*Pseudomonas aeruginosa* PAO1]

MLLGLSRSLWGYRGFVLGSVKREFQSRYRGSLFGALWTVLNPLSMIVVYTVIFSQVMRARLPGVDDGLAY

SVYLCAGLLTWGLFAEITSRSQSMFIENANLLKKISFPRICLPVIVLLNAGVNFAIILALFLGFLALSGR

LPGAALLALVPLLAIQVLFAAGLGMILGVLNVFFRDVGQLFGICLQFWFWLTPIVYPIGILPEGIRSLIE

LNPMTALMRSYQQLFLHGQWPDWPSLLPITLLALLLCALGLRLFRQRVGEMVDEL

>AAG06541.1 O-antigen translocase [*Pseudomonas aeruginosa* PAO1]

MSAAFINRVARVLVGTLGAQLITIGVTLLLVRLYSPAEMGAFSVWLSFATIFAVVVTGRYELAIFSTREE

GELQAIVKLILQLTLLIFVAVAIAVVIGRHLIESMPVVIGEYWFALAVASLGLGINKLVLSLLTFQQSFN

RLGVARVSLAACIAVAQVSAAYLLEGVSGLIYGQLFGVVVATALAALWVGKSLILNCIETPWRMVRQVAV

QYINFPKFSLPADLVNTVASQVPVILLAAKFGGDSAGWFALTLKIMGAPISLLAASVLDVFKEQAARDYR

EFGNCRGIFLKTFRLLAVLALPPFIIFWFIGEWAFGLVFGEAWAESGRYAVLMVPLFYMRFVVSPLSYTI

YIAQRQSMDLLWQLALLLLTFICFTLPDSVDSVLWFYSIAYAVMYFVYFWMSFQCAKGDAK

BlastP query sequence for Fig 3.

>ANE74943.1 permease [*Dickeya solani* IPO 2222]

MGTSYMLNDLKSALKNPESWVVLSWYDIKQRYKRSTLGPFWVTISTAILVGMLSLLWSTLFKLDVKDYLP

FFCIGQVFWTYISTQLTEASNGFIQFDYIIRQSKISFTSIMLRILSRNIIVFLHNFIIIIFVITFVGPGW

TFTALLSIIGFVLLSVALVSSSLILGVICTRFRDMQMIIQNILLVSFYFTPIMWKTDQLNEQWLYWVQFN

PLVHFFNIIREPMLGHLPDQNSIIIATAITVVLFILSMITLNKTKTKIAYWL

BlastP query sequence for Fig 5.

>ANE75210.1 enterobacterial common antigen polymerase [*Dickeya solani* IPO 2222]

MTEWAFFGLWLVWLGGGGVVLWLSGREFRRWRFNFNVLFSLLYLLTFYFGFPLTALLTFRFGVETASPLN

LLLALLSATAFYVIYYVSYKTRLRSAPPVSRPLPLTMTRLETLLTAGLLALVALVTAAVFFANNGLLLFK

LSSYSQIFSRDVAGVALKRFFYFFIPAMLMWYFLHQTRRAWLIFLLTTVAFGGLTYLLVGGTRANIIIAF

ALFLFIGLQRGWIAWWMLAAAGGGGIVAMFWLALKRYGLDVQGEEAFYTFLYLTRDTFSPWENLAMLWQH

LDQITLQGLAPIVRDFYVFIPAWLWPERPALVLNSANYFTWEVLNYHAGLAISPTLLGSLLIMGGPLLIP

VGAVAVGLLIKGFDGLYRYGRQAENRYLSAVLQAFCFGAVFNLIVLVREGLDAFVSRVVFFCLVFAACLL

AAKLLYWLLLRAGAVAVRAPSQGEKE

>ANE75212.1 O-antigen translocase [Dickeya solani IPO 2222]

MSLARASLWTAASTLVKIGAGLAVIKLLAVTFGPQGVGLAGNYRQLITVLGVMAGAGIANGVTRAVAAAP

PDANRSGPLLGTAVSLSMGCSLLLTLALWLLAAPLSRLLFGDDAYQPAIRALAWLQLGIAGASLLLAILK

GYQDARGNALAVMAGSLVGAVAYGASVWLGAYTGALVGLALMPALVCVPALILFFRRTPLGLRALTPDWS

WPLAGQLTRFSLMTLITAVTLPVGYVMMRNLLATHYTWQEVGVWQGVTTISDAWLQFITASFTVYLLPAL

ARLQDKRLVRQEILSALRFVLPVAATVGAAIWLLRDVAIHLLFSSAFSAMRDLFAWQLAGDVLKVGAYVF

GYLVVARASLRFYLLAELGQFLLLTGFSRWLIPLHGALGASQAYLATYAVYFLLCCGVFILYCRRA

>ANE75213.1 dTDP-4-amino-4,6-dideoxygalactose transaminase [Dickeya solani IPO 2222]

MIPFNSPPVVGSELEYMQAAMRSGKLSGDGAFTRRCQQWLEHYSGSCNVLLTPSCTASLEMAALLLNIQP

GDEVIMPSYTFVSTANAFVLRGATIVFVDIRPDTLNLDENRIEAAITNKTRAIVVVHYAGVGCDMNAVMA

LARQYGLFVVEDAAQGMMSRYQERPLGAIGHIGCFSFHETKNYTAGGEGGATLINDPELVARAEIIREKG

TNRSQFFRGQADKYTWRDIGSSYLMADIQAAYLWGQLEAAQRIHERRLTLWQHYARAFAPLAAAGRATLP

VIPADCRHNGHLFFLRLRDEAERSAFIRHMKEAEILTVFHYIPLHSSPAGRQFGRFVGEDRHTTRESERL

VRLPLFYNLSDLDQRTVINSALSFFS

BlastP query sequence for Fig 6.

>ANE75628.1 lipopolysaccharide biosynthesis protein [*Dickeya solani* IPO 2222]

MSRQRLSVVLISHNAAELLPDCLASVDWADEIIVLDSGSSDDTLDVARRLGAQVYQNTDWPGFGKQRQLA

QQYAGGDYIFMIDTDERVTPALRQSIEATLESPESDAVYRCARRNLFLGRFMRHSGWYPDEVIRLYPNRY

RYNDNAVHESLDYGAARVISLDGDLKHLTCRDFFSFQQKQFAYAESWAIERFRQGKRCGFAAIVLHTLGA

FVKTWLLRAGFLDGKQGLLLAIVNAQYTFNKYTGLWALNNRRDTHQEHQHHED

>ANE77768.1 3-deoxy-D-manno-octulosonic acid transferase [*Dickeya solani* IPO 2222]

MLQTLYTFLFYMIQPLIWLRLWLRGRKIPAYRKRWGERYGFYKNQVKPEGILLHSVSVGETLAAVPLVRA

LRHRYPSLPITVTTMTPTGSERALSAFGKDVYHVYLPYDLPGAMARFLDHVQPRLVIIMETELWPNLITA

LHQRKIPLIIANARLSERSANGYRKLGRFMRTLLRRITLIAVQNAEDGERFINLGLKRTQLNVTGSLKFD

ISVTPELAARAVTLRRQWAPQRPVWIAASTHEGEEKIIVDAHTELLKAFPTLLLILVPRHPDRFDDAKAI

VRKAGLEYTLRSAGTVPPASSHVVIGDSMGELMLLYGIADLAFVGGSLIERGGHNPLEPAAHAIPVLMGP

HTFNFKDICARLQESDGLITVRDTASLVEQVTTLLSDDDYRRYHGHHAVDVLHKNQGALQSLLALLEPYL

PPRSQ
